# Coverage with Tetanus, Diphtheria, and Acellular Pertussis Vaccine and Influenza Vaccine Among Pregnant Women — Minnesota, March 2013–December 2014

**DOI:** 10.15585/mmwr.mm6602a4

**Published:** 2017-01-20

**Authors:** Alexandra Barber, Miriam Halstead Muscoplat, Anna Fedorowicz

**Affiliations:** 1 Minnesota Department of Health, Division of Infections Disease, Epidemiology, Prevention and Control.

Pertussis and influenza infections can result in severe disease in infants. The diphtheria, tetanus, acellular pertussis (DTaP) vaccine is recommended for infants beginning at age 2 months, and influenza vaccine is recommended for infants aged ≥6 months. Vaccination of pregnant women induces the production of antibodies that are transferred across the placenta to the fetus and provide passive protection until infants are old enough to receive DTaP and influenza vaccines (*1*–*3*). To protect young infants before they are age-eligible for vaccination, the Advisory Committee on Immunization Practices (ACIP) has recommended since 2004 that all women who are or will be pregnant during influenza season receive inactivated influenza vaccine (*1*), and since 2013 that all pregnant women receive the tetanus, diphtheria, acellular pertussis (Tdap) vaccine (*3*). Tdap and influenza vaccination coverage was assessed among pregnant women in Minnesota. Vital records data containing maternal demographic characteristics, prenatal care data, and delivery payment methods were matched with vaccination data from the Minnesota Immunization Information Connection (MIIC) to assess vaccination coverage. MIIC stores vaccination records for Minnesota residents. Overall, coverage with Tdap vaccine was 58.2% and with influenza vaccine was 45.9%. Coverage was higher for each vaccine among women who received adequate prenatal care compared with those who received inadequate or intermediate care, based on the initiation of prenatal care and the number of recommended prenatal visits attended. Coverage also varied based on mother’s race, country of birth or region, and other demographic characteristics. Further study is needed to better understand the maternal vaccination disparities found in this study and to inform future public health initiatives.

Tdap and influenza vaccination coverage was assessed among women in Minnesota who had delivered a live birth during March 2, 2013–December 31, 2014. The beginning date was selected because it occurred 1 week after the most recent change in Tdap recommendations for pregnant women, providing an opportunity for most women included in the assessment to be vaccinated before delivery. Records for every live birth in Minnesota during March 2, 2013–December 31, 2014 were obtained from the Minnesota Department of Health’s Office of Vital Records. Demographic characteristics, including mother’s race, ethnicity, birth country, participation in the Special Supplemental Nutrition Program for Women Infants and Children (WIC) program, marital status, education level, gestational duration, prenatal care adequacy (assessed using the Kotelchuck Index*), and delivery payment methods were abstracted from the birth record. The Kotelchuck Index score was computed using vital records information on when prenatal care began and how many prenatal care visits were attended (*4*). Assessment was performed on the rate of receipt of ≥1 doses of Tdap vaccine and ≥1 doses of influenza vaccine during pregnancy among women in this cohort.

Using vital records data, a list of Minnesota women who delivered a live birth during March 2, 2013–December 31, 2014 was compiled and pregnancy intervals were calculated. This list was matched by mother’s name and birthdate to MIIC records. Tdap and influenza vaccinations during pregnancy were assessed. Frequencies, percentages, and risk ratios were determined for all demographic characteristics. Minnesota has a large Somali-born population; therefore, this group was analyzed separately from women born in all other African countries. Chi square tests and t-tests were used to test for significance. Because of the size of the cohort, statistical significance was set at p<0.001. Among women who received Tdap vaccine during their pregnancy, the percentage vaccinated during the optimal recommended time frame for vaccination (27–36 weeks gestation) (*3*) was also determined. The study was reviewed and approved by the University of Minnesota’s Institutional Review Board.

Among 127,073 live births in Minnesota with available and complete vital records for the period March 2, 2013–December 31, 2014, a total of 113,730 (89.5%) were matched to MIIC records. Among these women, 66,222 (58.2%) had received at least one Tdap vaccine, and 52,248 (45.9%) had received at least one influenza vaccine during pregnancy (Table 1). Among women who received Tdap vaccine, 57,215 (86.4%) were vaccinated during the recommended period (27–36 weeks gestation).

**TABLE 1:** Tdap and influenza vaccination coverage among pregnant women, based on vital records data and immunization records — Minnesota, March 2, 2013–December 31, 2014

Characteristic	Total study population	Received Tdap vaccination during pregnancy	Received Influenza vaccination during pregnancy
	No. (%)	No. (%)	No. (%)
**Overall**	**113,730**	**66,222 (58.2)**	**52,248 (45.9)**
**Maternal race**			
White	88,209 (77.6)	51,765 (58.7)	41,362 (46.9)
Black	12,192 (10.7)	6,785 (55.7)	4,756 (39.0)
American Indian	2,174 (1.9)	1,025 (47.2)	852 (39.2)
Asian Indian	1,658 (1.5)	1,020 (61.5)	796 (48.0)
Asian	6,879 (6.1)	4,124 (60.0)	3,259 (47.4)
Other	2,618 (2.3)	1,503 (57.4)	1,223 (46.7)
**Maternal ethnicity**			
Non-Hispanic	107,716 (94.7)	62,897 (58.4)	49,559 (46.0)
Hispanic	6,014 (5.3)	3,325 (55.3)	2,709 (45.0)
Maternal birth country/region*			
United States	95,889 (84.3)	56,497 (58.9)	44,833 (46.8)
Africa (excluding Somalia)	6,750 (5.9)	3,424 (50.7)	2,494 (37.0)
Somalia	3,402 (3.4)	1,370 (40.3)	1,370 (40.3)
Western Europe/Canada	974 (0.9)	508 (52.2)	392 (40.3)
Asia	6,657 (5.9)	3,896 (58.5)	3,053 (45.9)
Central and South America/Mexico	2,460 (2.2)	1,473 (59.9)	1,209 (49.2)
Eastern Europe	787 (0.7)	303 (38.5)	185 (23.5)
Other	165 (0.2)	99 (60.0)	59 (35.8)
**Mother’s education level**			
<High school diploma or GED	10,074 (9.0)	5,352 (53.1)	4,169 (41.4)
High school diploma or GED	18,665 (16.6)	10,476 (56.1)	8,061 (43.2)
<4 yrs college	22,158 (19.7)	12,781 (57.7)	9,666 (43.6)
Bachelor’s/Associate’s	46,688 (41.5)	27,879 (59.7)	22,341 (47.9)
Master’s/PhD/ professional	14,878 (13.2)	9,284 (62.4)	7,669 (51.6)
**Marital status**			
Married	77,135 (68.0)	44,287 (57.4)	35,567 (46.1)
Not married	36,281 (32.0)	21,927 (60.4)	16,673 (47.0)
**Payment**			
Private	74,053 (65.5)	44,559 (60.2)	35,714 (48.2)
Military	1,114 (1.0)	673 (60.4)	505 (45.3)
Uninsured	2,499 (2.2)	781 (31.3)	661 (26.5)
Medical assistance	33,629 (29.7)	19,111 (56.8)	14,460 (43.0)
Other	1,778 (1.6)	1,014 (57.0)	845 (47.5)
**Adequacy of prenatal care^†^**			
Adequate	87,094 (76.6)	53,281 (61.2)	42,314 (48.6)
Intermediate	13,241 (11.6)	7,079 (53.5)	5,759 (43.5)
Inadequate	13,395 (11.8)	5,862 (43.8)	3,700 (32.3)
**Received WIC**			
Yes	36,700 (32.6)	21,268 (58.0)	16,543 (45.1)
No	76,014 (67.4)	44,685 (58.8)	35,488 (46.7)

**Abbreviations**: GED = general educational development certificate; Tdap = tetanus, diphtheria, acellular pertussis vaccine; WIC = Special Supplemental Nutrition Program for Women, Infants, and Children.

*Missing values for maternal birth country (n = 48), education (n = 1,267), marital status (n = 314), payment (n = 657), WIC status (n = 1,016).

^†^ Based on the Kotelchuck Index, which considers time of initiation of prenatal care and number of prenatal visits attended.

Unadjusted risk ratios for Tdap and influenza vaccinations were calculated across selected demographic characteristics. Tdap and influenza vaccination coverage rates were significantly lower among pregnant women who received inadequate or intermediate prenatal care compared with women who received adequate prenatal care. Rates were also significantly lower among black and American Indian women when compared with white women, and among women born in Africa (particularly Somalia), Eastern Europe, Western Europe, and Canada compared with women born in the United States. In addition, vaccination coverage was lower among Hispanic women than non-Hispanic women (for Tdap only), women with lower levels of education, and women who were receiving medical assistance, or were uninsured (Table 2).

**TABLE 2:** Unadjusted relative risks for Tdap and influenza vaccination during pregnancy by selected demographic characteristics — Minnesota births, March 2, 2013–December 31, 2014

Characteristic	Tdap vaccination unadjusted relative risk % (95% CI)	Influenza vaccination unadjusted relative risk % (95% CI)
**Maternal race (referent = white)**		
Black	0.95* (0.93–0.96)	0.83* (0.81–0.85)
American Indian	0.80* (0.77–0.84)	0.84* (0.79–0.88)
Asian Indian	1.05 (1.01–1.09)	1.02 (0.97–1.08)
Asian	1.02 (1.00–1.04)	1.01 (0.98–1.04)
**Maternal ethnicity (referent = non-Hispanic)**		
Hispanic	0.95* (0.93–0.97)	0.98 (0.95–1.01)
**Maternal birth country/region (referent = United States)**		
Africa (excluding Somalia)	0.86* (0.84–0.88)	0.79* (0.77–0.82)
Somalia	0.68* (0.66–0.71)	0.58* (0.55–0.61)
Western Europe/Canada	0.89* (0.83–0.94)	0.86* (0.80–0.93)
Asia	0.99 (0.97–1.01)	0.98 (0.95–1.01)
Central and South America/Mexico	1.02 (0.98–1.05)	1.05 (1.01–1.09)
Eastern Europe	0.65* (0.60–0.71)	0.50* (0.44–0.57)
**Mother’s education level (referent = bachelor’s/associate’s degree)**		
<High school diploma or GED	0.89* (0.87–0.91)	0.86* (0.84–0.89)
High school diploma or GED	0.94* (0.93–0.95)	0.90* (0.89–0.92)
<4 yrs college	0.97* (0.95–0.98)	0.91* (0.90–0.93)
Master’s/PhD/professional degree	1.05* (1.03–1.06)	1.08* (1.06–1.10)
**Married (referent = yes)**		
No	1.05* (1.04–1.06)	1.00 (0.98–1.01)
**Payment (referent = private insurance)**		
Military	1.00 (0.96–1.03)	0.94 (0.88–1.00)
Uninsured	0.52* (0.49–0.55)	0.55* (0.51–0.59)
Medical assistance	0.94* (0.93–0.95)	0.89* (0.88–0.90)
**Prenatal care**^†^ **(referent = adequate)**		
Intermediate	0.87* (0.86–0.89)	0.90* (0.88–0.91)
Inadequate	0.71* (0.70–0.73)	0.67* (0.65–0.68)
**Received WIC (referent = no)**		
Yes	1.01 (1.00–1.03)	1.04* (1.02–1.05)

**Abbreviations**: GED = general educational development certificate; Tdap = tetanus, diphtheria, acellular pertussis vaccine; WIC = Special Supplemental Nutrition Program for Women, Infants, and Children.

* p <0.001.

^†^ Based on the Kotelchuck Index, which considers time of initiation of prenatal care and number of prenatal visits attended.

## Discussion

*Healthy People 2020* goals include decreasing the number of infant pertussis cases by 10% and increasing influenza vaccination coverage among pregnant women (*5*). Although it is not possible to assess progress on the infant pertussis goal using only maternal Tdap vaccination data, influenza vaccination coverage of 46% among pregnant women in Minnesota is well below the overall *Healthy People 2020* goal of 70% of adults aged ≥18 years receiving seasonal influenza vaccine (*5*). Suboptimal coverage levels might be related to the quality of prenatal care and concerns about vaccine safety during pregnancy. One study found that women who were generally supportive of vaccines expressed concern over vaccines given during pregnancy (*6*). Another study found that 67% of patients accepting the monovalent influenza 2009 H1N1 vaccine said their obstetrician’s recommendation was a major factor in their decision (*7*). According to an Internet panel survey conducted during March–April 2014, influenza vaccination coverage among pregnant women who received a provider recommendation and offer for influenza vaccination was more than twice as high as coverage among women who received a recommendation but no offer, and seven times higher than coverage among women who received no recommendation and no offer (*8*).

This study demonstrates demographic disparities in Tdap and influenza vaccination coverages among pregnant women in Minnesota, including by race, maternal birth country or region, maternal educational attainment, insurance coverage at delivery, and adequacy of prenatal care. Additional studies are needed to identify barriers to vaccination faced by women in different demographic groups to inform the development of effective strategies to address these disparities.

The findings in this report are subject to at least four limitations. First, because submitting immunization data to MIIC was not required for health care providers in Minnesota at the time of this study, some MIIC records might be incomplete and some Minnesota residents might not be in MIIC. Therefore, actual vaccine coverage might be different than results suggest. Second, self-reported demographic data and inconsistent reporting of prenatal care data across different health care facilities might result in data misclassification. The Office of Vital Records data are self-reported by birth parents, with the exception of prenatal care data, which are completed by health care facilities. Misclassification could potentially result in artificial demographic disparities. Third, the start date of the study was 1 week after publication of the current ACIP recommendation for Tdap vaccination during pregnancy. Because it takes time for health care providers to become familiar with and begin implementing new vaccine recommendations, it is likely that initial coverage rates were low because prenatal care providers were unaware of the new recommendation, or because their clinical practice guidelines had not yet been updated. Although rates might have been lower at the beginning of the study period for these reasons, from 2013 to 2014, Tdap vaccination coverage during pregnancy increased 16.8%, suggesting that more prenatal care providers adopted the new recommendation as they became aware of it. Finally, this study assessed whether a pregnant woman received at least one influenza vaccine during her pregnancy. If a pregnancy spanned two influenza seasons, women might have only received one vaccine, which would not provide optimal protection because of annual strain selection changes made to the vaccine. Therefore, this study might not accurately represent protection against the circulating influenza strains among pregnant women.

Studies have demonstrated the positive impact of a strong provider recommendation for vaccination (*6*–*8*); however, more information is needed to understand the factors that influence strong recommendations, such as the time and training required to adopt and implement them. Addressing these factors will help providers make strong vaccine recommendations during prenatal care visits. Future studies also are needed to assess the timing of influenza vaccination during pregnancy to better understand whether pregnant women are being appropriately vaccinated over consecutive influenza seasons. In addition, further investigation into reasons for lower vaccination coverage among certain racial and ethnic groups need to be explored to assist public health professionals and clinicians in addressing community-specific barriers to maternal vaccination.

**Summary**

**What is already known about this topic?**

The Advisory Committee on Immunization Practices recommends that women who are or will be pregnant during the influenza season be vaccinated with inactivated influenza virus vaccine, and that all pregnant women receive a dose of tetanus, diphtheria, acellular pertussis (Tdap) vaccine in every pregnancy. Vaccination during pregnancy protects infants from influenza and pertussis during the first year of life through passively acquired maternal antibodies.

**What is added by this report?**

Among 113,730 women in Minnesota who had delivered a live birth during March 2013–December 2014 and for whom immunization records were available, 58% received a Tdap vaccination and 46% received an influenza vaccination during their pregnancy. Tdap and influenza coverage rates were significantly lower among pregnant women who received inadequate or intermediate prenatal care; black and American Indian women; women born in Africa (particularly Somalia), Eastern Europe, and Western Europe or Canada; women with lower levels of education; and women who were receiving medical assistance or who were uninsured, than among women who received adequate prenatal care, were white, U.S.-born, had higher levels of education, and had private insurance.

**What are the implications for public health practice?**

Measures are needed to improve adequacy of prenatal care and reduce health disparities among minority, poor, and non–U.S.-born women to address maternal vaccination disparities.

## Footnotes

* The Kotelchuck Index considers adequate prenatal care as the initiation of prenatal care by the 4th month of pregnancy and attendance at ≥80% of prenatal care visits recommended by the American College of Obstetricians and Gynecologists (ACOG). Intermediate prenatal care is considered the initiation of prenatal care by the 4th month of pregnancy and attendance at ≥50% of recommended visits. Inadequate prenatal care is considered as starting prenatal care after the 4th month of pregnancy or attendance at <50% of recommended visits. This analysis considered all pregnancies to be normal and did not account for groups at high risk. The Kotelchuck Index also considered prenatal care to be adequate plus if >110% of visits were attended, but this level of care was not included in the analysis.
